# Multimodal neuroimage data fusion based on multikernel learning in personalized medicine

**DOI:** 10.3389/fphar.2022.947657

**Published:** 2022-08-17

**Authors:** Xue Ran, Junyi Shi, Yalan Chen, Kui Jiang

**Affiliations:** Department of Medical Informatics, Nantong University, Nantong, China

**Keywords:** neuroimaging, personalized medicine, multimodal data fusion, multikernel learning, magnetic resonance imaging, positron emission tomography

## Abstract

Neuroimaging has been widely used as a diagnostic technique for brain diseases. With the development of artificial intelligence, neuroimaging analysis using intelligent algorithms can capture more image feature patterns than artificial experience-based diagnosis. However, using only single neuroimaging techniques, e.g., magnetic resonance imaging, may omit some significant patterns that may have high relevance to the clinical target. Therefore, so far, combining different types of neuroimaging techniques that provide multimodal data for joint diagnosis has received extensive attention and research in the area of personalized medicine. In this study, based on the regularized label relaxation linear regression model, we propose a multikernel version for multimodal data fusion. The proposed method inherits the merits of the regularized label relaxation linear regression model and also has its own superiority. It can explore complementary patterns across different modal data and pay more attention to the modal data that have more significant patterns. In the experimental study, the proposed method is evaluated in the scenario of Alzheimer’s disease diagnosis. The promising performance indicates that the performance of multimodality fusion *via* multikernel learning is better than that of single modality. Moreover, the decreased square difference between training and testing performance indicates that overfitting is reduced and hence the generalization ability is improved.

## 1 Introduction

Neuroimaging technologies are currently the most widely used methods to study brain diseases, and they can directly or indirectly image the nervous system. Common neuroimaging techniques include structural magnetic resonance imaging (sMRI), which can provide rich morphological features of brain tissues; functional magnetic resonance imaging (fMRI), which not only provides anatomical information but also shows the response mechanism of the nervous system; positron emission tomography (PET), which is the only novel imaging technique that can display biomolecular metabolism, receptors, and neuromediator activity *in vivo*; diffusion tensor imaging (DTI), which can reflect the structure of white matter fibrin in the brain, etc (Klöppel et al., 2012; Friston, 2009). Neuroimaging technologies play a very important role in the research of Alzheimer’s disease (AD) (Bao et al., 2021; Karikari et al., 2021; Zhang et al., 2021). Previous studies on AD and mild cognitive impairment (MCI) were often based on a single neuroimaging technique (single modality data). However, single modality data have obvious defects; they can only provide information on local brain abnormalities, which will affect the diagnostic accuracy of AD and MCI. In recent years, many studies have found that multimodal data have the advantage of realizing information complementation (Zhang et al., 2022a). The features of multimodal data can be combined to obtain more comprehensive disease information, which is of great significance for the early diagnosis and treatment of AD. In particular, with the development of artificial intelligence (AI) technologies, multimodal fusion has been developed rapidly for AD diagnosis studies. For example, Kohannim et al. (2010) used support vector machines (SVMs) to classify AD. When using MRI as single-modal data for experiments, the classification accuracy of AD vs. normal control (NC) and that of MCI vs. NC were 79.07% and 71.21%, respectively. When experiments were performed after combining MRI, fluorodeoxyglucose-PET, and cerebrospinal fluid (CSF), the classification accuracy of AD vs. NC and that of MC vs. NC were 90.70% and 75.76%, respectively. Compared to single modality, the classification accuracy is improved by 5–10%. Zhang et al. (2011) combined MRI, PET, and CSF for AD classification. A multikernel SVM was taken as the classifier. The classification accuracy of AD vs. NC was 93.2%. Compared with using single-modal data, the accuracy was improved by 7–10%. The accuracy of MCI vs. NC was 76.4%, which was an improvement of 4.4–5% compared to using single modality data. Buvaneswari and Gayathri (2021) combined the features extracted from DTI and fMRI into a multikernel SVM for AD classification, and the accuracy of AD vs. NC was 98.4%; however, when the two modalities were used alone for classification, the highest achieved accuracy was only 90.9%. The above research further verifies that in the classification of AD, compared with single-modal data, the use of multimodal data can obtain richer and more valuable features, and the classifier can obtain higher classification accuracy.

From existing studies regarding multimodality fusion, we found that classifiers based on multikernel learning were commonly used. This is because each modality can be mapped into the kernel space by a kernel function. Therefore, multikernel learning actually provides a natural framework for multimodality fusion. However, when multikernel learning is applied to practice, e.g., medical data analysis, overfitting often exists. Therefore, to overcome overfitting and to obtain promising prediction performance, in this study, according to regularized label relaxation linear regression (Fang et al., 2017), we integrate label relaxation and compactness graph mechanisms into multikernel learning and propose a new multikernel learning algorithm for AD diagnosis.

The main differences with the existing studies can be summarized as follows.

(1) Unlike the modality-consistent regularization used in previous studies (Jiang et al., 2016), the “all-single” fusion strategy is introduced so that every single feature and the possible combinations are all considered so that the complementary information can be fully explored.
(2) We extend the compactness graph mechanism from the linear space to the multikernel space so that the overfitting problems can be reduced in the multikernel space.

The remaining article is organized into four sections. In Section 2, we will state some related work regarding AI-assisted AD diagnosis based on multimodality fusion. In Section 3, we will present our new method and its optimization. In Section 4, we will report our experimental results and in the last, we will conclude our study and indicate our future work.

## 2 Related work

Multimodality fusion strategies can be divided into three levels: pixel-level fusion, feature-level fusion, and decision-level fusion (Xia et al., 2020). Pixel-level fusion is to directly perform pixel-related fusion based on strict registration. Feature-level fusion refers to transforming different modal data into high-dimensional feature spaces and then merging them before or during modeling. Decision-level fusion is to use certain strategies, such as voting, to fuse the decision result of each modal, to obtain the globally optimal result. In Table 1, we summarize some representative previous works belonging to these three categories.

**TABLE 1:** Representative works of multimodality fusion.

Categories	Authors	Modalities	Methodologies
Pixel-level	Daneshvar and Hassan, (2010)	MRI, PET	A model based on integrated intensity-hue-saturation and retina-inspired model was proposed to improve the fusion performance
	Li and Wang, (2012)	SPECT, MRI	A method of multiscaled combination of MR and SPECT images based on variable-weight
	Bhatnagar et al. (2015)	MRI and PET	A novel framework for spatially registered multimodal medical image fusion based on nonsubsampled contourlet transform
Decision-level	Dimitriadis et al. (2018)	MRI	A random forest feature selection, fusion, and ensemble strategy was applied to the classification and prediction of AD
	Fan et al. (2008)	MRI and PET	An SVM-based ensemble method was proposed and two modal data of the bilateral hippocampus volume and the bilateral entorhinal cortex volume as core features were used for AD prediction
	Zeng et al. (2018)	sMRI, PET, and CSF	An SVM-based ensemble method was proposed and the combined features of sMRI, PET, and CSF were used to build an ensemble classification model for AD prediction
Feature-level	Zhang et al. (2020)	MRI and PET	A deep multimodal fusion network based on an attention mechanism, which was able to selectively extract deep features from MRI and PET was proposed to predict AD
	Suk et al. (2014)	MRI and PET	High-level latent and shared feature representations were extracted and fused from neuroimaging *via* deep network-confined Boltzmann machines
	Madusanka et al. (2019)	MRI and PET	Texture and morphological features were fused as a biomarker to diagnose AD. SVM was taken as the classifier

Strict registration plays a key role in pixel-level fusion. For example, Daneshvar et al. proposed a fusion strategy based on integrated intensity-hue-saturation and retina-inspired model to improve the fusion performance. The strategy often used in decision-level fusion is ensemble learning. In the early studies of AD diagnosis, the most commonly used learning components in ensemble learning were SVM (Shukla et al., 2020) and also linear classifiers (Jiang et al., 2020), Bayesian networks (Zhang et al., 2017), decision trees (Zhang et al., 2020), etc. For example, Fan et al. (2008) took the two-modal data of the bilateral hippocampus volume and the bilateral entorhinal cortex volume as core features and used SVM as the learning component. The accuracies of AD vs. MCI, AD vs. NC, and MCI vs. NC are 58.30%, 82.00%, and 76.00% respectively.

Feature-level fusion has been widely used in AD studies. For example, Suk et al. (2014) obtained high-level latent and shared feature representations from neuroimaging *via* deep network-confined Boltzmann machines. In the binary classification of AD vs. NC and MCI vs. NC, maximum accuracies of 95.35% and 85.67% were finally obtained, respectively. Madusanka et al. (2019) used the fusion of texture and morphological features as a biomarker to diagnose AD and used SVM as the classifier. The classification accuracy reached 86.61%. Zhang et al. (2020) proposed a deep multimodal fusion network based on an attention mechanism, which was able to selectively extract deep features from MRI and PET, while suppressing irrelevant information. In the attention mechanism-based model, the fusion ratio of each modality is automatically assigned according to the importance of the modality. In addition, a hierarchical fusion method was adopted to ensure the effectiveness of multimodal data fusion. The final classification accuracies of NC vs. AD and SMCI vs. PMCI were 95.21% and 89.79%, respectively.

In this study, we also focus on feature-level fusion. From previous studies regarding feature-level fusion, we find that there are still some issues that should be addressed in the future.

(1) Most of the previous studies only direct concatenate features from different modalities and then input them into a model for AD prediction. This strategy does not consider complementary patterns across different modalities.
(2) Some multikernel-based studies achieved promising performance and also consider complementary patterns across different modalities. However, with a sparse or small training set, overfitting often occurs.

Therefore, to address the abovementioned issues, in this study, we will propose a novel multimodality fusion model at the feature-fusion level.

## 3 Data and methods

### 3.1 Data

**FIGURE 1:**
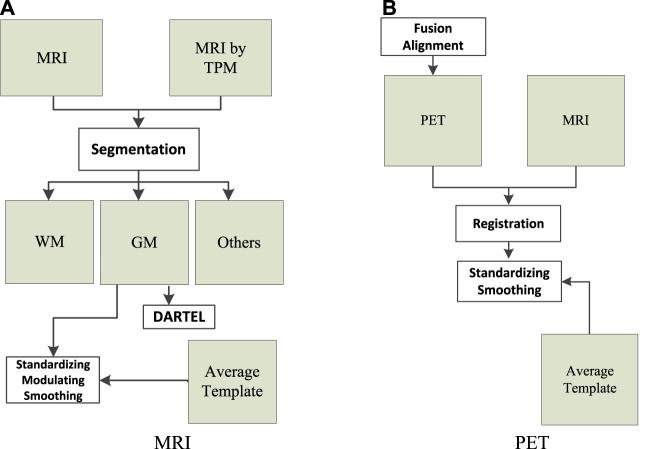
Data preprocessing: **(A)** magnetic resonance imaging (MRI) and **(B)** positron emission tomography (PET).

The data (MRI and PET) used in this study were collected from Alzheimer’s Disease Neuroimaging Initiative. There are 103 subjects in the dataset, where 51 subjects were organized into the NC group and 52 subjects were organized into the AD group. We used the following workflows (Zhang et al., 2021), as shown in Figure 1, to perform data preprocessing.

As can be seen from Figure 1A, the tissue probability map template was first used to segment the original MRI into white matter (WM), gray matter (GM), and other tissues. In particular, WM and GM tissues were mapped into the Montreal Neurological Institute (MNI) space during preprocessing. Second, diffeomorphic anatomical registration through exponentiated lie algebra (DARTEL) was employed to create average templates for the obtained WM and GM tissues. In the last, GM was modulated to transform the density information into volume information. In addition, GM was smoothed (8 mm Gaussian) to avoid the influences caused by noises.

As can be seen from Figure 1B, SPM-12 was employed to fuse these PET images (one subject has 96 images) to construct a 3-D image that provides brain spatial information and the feature information between tissue structures was also retained. Moreover, head motion was corrected. After fusion alignment, MRI and PET of each subject were registered and affinely aligned. In the last, the average template data generated in Figure 1A were used to spatially normalize all PET images to the standard MNI space. PET images were also smoothed (8 mm Gaussian) to avoid the influences caused by noises.

### 3.2 Methods

#### 3.2.1 Kernelized regularized label relaxation

A regularized label relaxation (RLR) linear regression model was proposed to address the overfitting problem (Fang et al., 2017). The objective function is defined as follows:

$$\begin{array}{l} \underset{A,M}{\min}{\left\|\mathrm{XA}-\left(Y+B⊙M\right)\right\|}_{F}^{2}+\lambda tr\left(A^{T}X^{T}\mathrm{LXA}\right)\\ s.t\;M\geq 0 \end{array}$$ (1)

where {X,Y} represents the training set, B represents a luxury matrix derived from Y , A represents the transformation matrix, M represents a nonnegative label relaxation matrix, L represents the Laplacian matrix, λ is a regularized parameter, tr() represents the trace of a matrix, and ⊙ is a Hadamard product operator. RLR can classify linear data well and restrain overfitting. However, in many real-world scenarios, especially in the medical field, many data are not linear, which may limit the application of RLR. Therefore, Fang et al. employed the kernel technique to further extend RLR to its nonlinear version, that is, kernelized RLR (KRLR). The objective function of KRLR is defined as follows:

$$\begin{array}{l} \underset{\Theta ,M}{\min}{\left\|\mathrm{K\Theta}-\left(Y+B⊙M\right)\right\|}_{F}^{2}+\lambda tr\left(\Theta^{T}K^{T}\mathrm{LK\Theta}\right)\\ s.t\;M\geq 0 \end{array}$$ (2)

where Θ can be considered the transformation matrix and the new K is a positive semidefinite kernel Gram matrix in which each element can be calculated as follows:

$$K_{ij}={\left[<\phi \left(X\right),\phi {\left(X\right)}^{T}>\right]}_{ij}=k\left(x_{i}^{T},x_{j}^{T}\right).$$ (3)

In Eq. 3, $\phi \left(X\right)=\left[\phi {\left(x_{1}\right)}^{T},\phi {\left(x_{2}\right)}^{T},...,\phi {\left(x_{N}\right)}^{T}\right]$ , $\phi :R^{d}\to \Gamma$ is a nonlinear function that maps the input data from the original feature space to the Hilbert space Γ . $k:R^{d}\times R^{d}\to R$ represents a kernel function in which the polynomial kernel, Gaussian kernel, and the hyperbolic tangle kernel are usually adopted.

#### 3.2.2 Multikernel kernelized regularized label relaxation

We know that multikernel learning provides us a natural framework for multimodal data fusion (Wang et al., 2021). Therefore, we can extend KRLR to its multikernel version by adjusting the generation way of the kernel Gram matrix. In this study, a linear combination is used to generate the new kernel Gram matrix in the multikernel space, that is,

$$K=\sum_{m=1}^{M}\alpha_{m}K_{m}.$$ (4)

By substituting Eq. 4 into Eq. 2, we can obtain the objective function of multikernel KRLR,

$$\begin{array}{l} \underset{\Theta ,M,\alpha_{m}}{\min}{\left\|\sum_{m=1}^{M}\alpha_{m}K_{m}\Theta -\left(Y+B⊙M\right)\right\|}_{F}^{2}+\lambda tr\left(\Theta^{T}{\left(\sum_{m=1}^{M}\alpha_{m}K_{m}\right)}^{T}L\left(\sum_{m=1}^{M}\alpha_{m}K_{m}\right)\Theta \right)\\ s.t\;M\geq 0,\sum_{m=1}^{M}\alpha_{m}=1 \end{array}.$$ (5)

In Eq. 5, three components are required to be optimized; they are the transformation matrix Θ , the relaxation matrix M , and the linear kernel combination coefficient $\alpha_{m}$ . Since the objective function in Eq. 6 is convex, an iterative updating strategy is adopted for optimization so that in each iteration a closed-form solution can be guaranteed (Xiang et al., 2012).

To devise the updating rule regarding the transformation matrix Θ , we suppose that the relaxation matrix M and the linear kernel combination coefficient $\alpha_{m}$ have been fixed; thus, the optimization problem becomes

$$J\left(\Theta \right)=\underset{\Theta }{\min}{\left\|\sum_{m=1}^{M}\alpha_{m}K_{m}\Theta -\left(Y+B⊙M\right)\right\|}_{F}^{2}+\lambda tr\left(\Theta^{T}{\left(\sum_{m=1}^{M}\alpha_{m}K_{m}\right)}^{T}L\left(\sum_{m=1}^{M}\alpha_{m}K_{m}\right)\Theta \right)$$ (6)

By setting the derivation of Eq. 6 with respect to the transformation matrix Θ to 0, that is, ∂J(Θ)/∂Θ=0 , we have

$$\Theta ={\left({\left(\sum_{m=1}^{M}\alpha_{m}K_{m}\right)}^{T}\left(\sum_{m=1}^{M}\alpha_{m}K_{m}\right)+\lambda {\left(\sum_{m=1}^{M}\alpha_{m}K_{m}\right)}^{T}L\left(\sum_{m=1}^{M}\alpha_{m}K_{m}\right)\right)}^{-1}{\left(\sum_{m=1}^{M}\alpha_{m}K_{m}\right)}^{T}\left(Y+B⊙M\right)$$ (7)

To devise the updating rule regarding the relaxation matrix M , we suppose that the transformation matrix Θ and the linear kernel combination coefficient $\alpha_{m}$ have been fixed; thus, the optimization problem becomes

$$\begin{array}{l} \underset{\Theta ,M,\alpha_{m}}{\min}{\left\|\sum_{m=1}^{M}\alpha_{m}K_{m}\Theta -\left(Y+B⊙M\right)\right\|}_{F}^{2}\\ s.t\;M\geq 0 \end{array}.$$ (8)

The solution of **M** can be finally obtained as follows:

$$M=\max\left(B,\sum_{m=1}^{M}\alpha_{m}K_{m}\;\Theta -Y\right).$$ (9)

To devise the updating rule regarding the kernel combination coefficient $\alpha_{m}$ , we suppose that the transformation matrix Θ and the relaxation matrix M have been fixed; thus, the optimization problem becomes

$$\begin{array}{l} J\left(\Theta \right)=\underset{\Theta }{\min}{\left\|\sum_{m=1}^{M}\alpha_{m}K_{m}\Theta -\left(Y+B⊙M\right)\right\|}_{F}^{2}+\lambda tr\left(\Theta^{T}{\left(\sum_{m=1}^{M}\alpha_{m}K_{m}\right)}^{T}L\left(\sum_{m=1}^{M}\alpha_{m}K_{m}\right)\Theta \right)\\ s.t\;\sum_{m=1}^{M}\alpha_{m}=1 \end{array}.$$ (10)

From Eq. 10, it can be seen that the analytical solution of $\alpha_{m}$ cannot be directly obtained. In this study, the reduced gradient method is used to obtain the optimal $\alpha_{m}$ (Rakotomamonjy et al., 2008). To be specific, when the gradient of Eq. 10 with respect to $\alpha_{m}$ is obtained, $\alpha_{m}$ can be updated along its decent direction $D_{m}$ to ensure that the equality constraint and the nonnegativity constraints on $\alpha_{m}$ are satisfied. Let $\alpha_{g}$ be a nonzero entry of α , then $\nabla_{reg}J$ , which represents the reduced gradient of Eq. 10, has components ${\left[\nabla_{reg}J\right]}_{m}$ and ${\left[\nabla_{reg}J\right]}_{g}$ that are defined as

$${\left[\nabla_{reg}J\right]}_{m}=\frac{\partial J}{\partial \alpha_{m}}-\frac{\partial J}{\alpha_{g}},\forall m\neq g$$ (11)

$${\left[\nabla_{reg}J\right]}_{g}=\sum_{m\neq g}\left(\frac{\partial J}{\partial \alpha_{g}}-\frac{\partial J}{\alpha_{m}}\right)$$ (12)

where g is the index of the largest element in **α**. The positivity constraints have also to be taken into account in the descent direction. However, if there is an index *m* such that $\alpha_{m}=\text{0}$ and ${\left[\nabla_{reg}J\right]}_{m}>0$ , using this direction would violate the positivity constraint for $\alpha_{m}$ . Hence, the descent direction for that component is set to 0. This gives the descent direction for update $D_{m}$ as

$$D_{m}=\{\begin{array}{cc} 0 & \text{if}\;\alpha_{m}>0\;\text{and}\;\frac{\partial J}{\partial \alpha_{m}}-\frac{\partial J}{\alpha_{g}}\text{>0}\\ -\frac{\partial J}{\partial \alpha_{m}}+\frac{\partial J}{\alpha_{g}} & \text{if}\;\alpha_{m}>0\;\text{and}\;m\neq g\\ \sum_{m\neq g}\left(\frac{\partial J}{\partial \alpha_{g}}-\frac{\partial J}{\alpha_{m}}\right) & \text{if}\;m\neq g \end{array}$$ (13)

### 3.3 Algorithm

Based on the solutions to the transformation matrix Θ , the relaxation matrix M , and the kernel combination coefficient $\alpha_{m}$ , detailed algorithm steps were deduced as follows.

Algorithm 1

Input: Multi-modal training data $\left\{x_{i}^{\left(m\right)}\text{,}y_{i}\right\}$ and the regularized parameter λ .
Output: Transformation matrix Θ , relaxation matrix M and kernel combination coefficient $\alpha_{m}$ Procedures:
Use “All-single” fusion strategy to obtain input data from $\left\{x_{i}^{\left(m\right)}\text{,}y_{i}\right\}$ . Initialize α by setting $\alpha_{m}=1\text{/}M$ .
Randomize M .
**Repeat**
  Update Θ by equation (7).
  Update M by equation (9).
  Update ${\partial J/\partial \alpha }_{m}$ and $D_{m}$ by equation (13).
  Update $g=\underset{m}{\arg\max}\alpha_{m}$ .
  Set $J^{†}=0,\alpha^{†}=\alpha ,D^{†}=D$ .
**Repeat**
  Update $\alpha =\alpha^{†},D=D^{†}$ .
  Update $v=\underset{\left\{m|D_{m}<0\right\}}{\arg\min}-\alpha_{m}/D_{m}$ .
  Update $\beta_{\max}=-\alpha_{v}/D_{v}$ .
  Update $\alpha^{†}=\alpha +\beta_{\max}D$ .
  Update $D_{m}^{†}=D_{m}-D_{v},D_{v}^{†}=0$ .
  Update $J^{†}$ by $\sum_{m=1}^{M}\alpha_{m}^{†}K_{m}$

**Until** ( $J^{†}\geq J$ )
**Until** (*convergence*)

The time complexity of Algorithm 1 consists of 3 parts: the computation of Θ , the computation of M , and the computation of α . From Eq. 7, it is easy to find that the time complexity of the computation of Θ is $O\left(N^{3}\right)$ , and from Eqs. 9 and 13, we see that the computation of M and α is $O\left(N^{2}\right)$ . Therefore, the asymptotic time complexity of Algorithm 1 is $O\left(N^{3}\right)$ .

## 4 Experimental results

### 4.1 Settings

The original features extracted from sMRI and PET images were represented in a very high-dimensional feature space. Therefore, the direct use of high-dimensional features for modeling will lead to the curse of dimensionality (Chandrashekar and Sahin, 2014). That is to say, samples become very sparse in the high-dimensional space, so the discriminability between samples will be significantly reduced. Therefore, before modeling, feature selection was performed to reduce the dimension of feature spaces. In this study, the *Fish score* was employed as the supervised method to reduce the irrelevant features to the outcome. In *Fish score*, we select the first 30 features with the highest-ranking values for the next unsupervised feature selection. *Person score* was employed as the unsupervised method to reduce the redundancy between features. In *Person score*, the threshold is set to 0.4.

**FIGURE 2:**
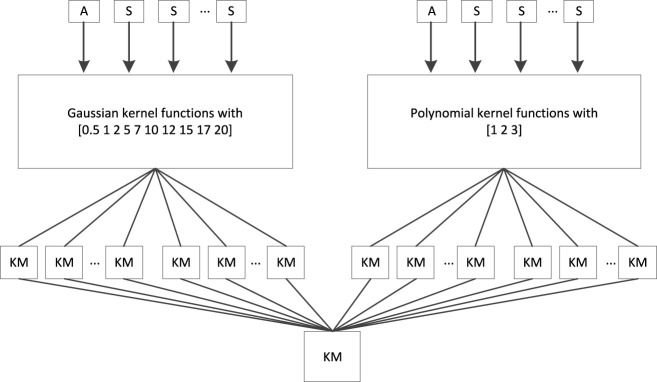
“All-single” fusion strategy.

**FIGURE 3:**
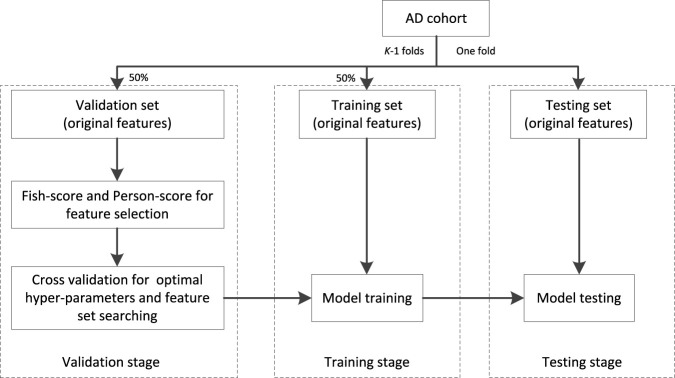
Workflow of training.

Regarding multikernel learning, the “all-single” strategy, as shown in Figure 2, was adopted to fuse sMRI features and PET features. In Figure 3, “A” represents the combined features of sMRI and PET, “S” represents each sMRI or PET feature, and “KM” denotes the kernel matrix. Suppose we had a dataset $\chi ={\left[x_{i1}^{\left(m\right)},x_{i2}^{\left(m\right)},x_{i3}^{\left(m\right)}\right]}_{i=1,2,3,4,m=1,2}$ having 3 subjects, each subject has two modalities (*m* = 1 and 2), and each modality has 4 features (*i* = 1, 2, 3, and 4), then “A” in Figure 2 can be expressed as ${\left[x_{i1}^{\left(1\right)},x_{i2}^{\left(1\right)},x_{id}^{\left(1\right)},x_{i1}^{\left(2\right)},x_{i2}^{\left(2\right)},x_{id}^{\left(2\right)}\right]}_{i=1,2,3,4}$ , and “S” can be expressed as ${\left[x_{i1}^{\left(m\right)},x_{i2}^{\left(m\right)},x_{i3}^{\left(m\right)}\right]}_{i=1,2,3,4,m=1,2}$ . According to Rakotomamonjy et al., (2008), {0.5, 1, 2, 5, 7, 10, 12, 15, 17, 20} is taken as a Gaussian kernel parameter candidate set and {1, 3, 5} is taken as a polynomial kernel parameter candidate set. Therefore, with such settings, 91 KMs were finally generated, and the goal of multikernel learning is to learn the coefficient of each KM.

The workflow chart of training is shown in Figure 3. The AD cohort is first partitioned into *K* (*K* = 5 in our study) folds, one is taken as the testing set and the remaining are taken as the validation set (50%) and training set (50%). At the stage of validation, the *Fish score* is employed as the supervised method to reduce the irrelevant features to the outcome. *Person score* is employed as the unsupervised method to reduce the redundancy between features. Then the cross-validation (5-CV) strategy is used to determine the optimal feature set and hyper parameters (the regularized parameter λ is searched from 0.0001 to 1) with respect to the proposed model. At the stage of training, with the optimal feature set and hyper parameters, the best model can be obtained. At the stage of testing, with the best model, we can obtain the corresponding testing results. The workflow shown in Figure 3 is repeated *K* times so that each fold has the opportunity to become the testing set.

To highlight the performance of our multimodality fusion method, a single modality model ridge regression (RR) and 4 multimodality fusion models, i.e., MV-TSK-FS (Jiang et al., 2016), simpleMKL (Rakotomamonjy et al., 2008), RFF-MKL (Liu et al., 2013), and MV-L2-SVM (Wang et al., 2015), are introduced for comparison study. Table 2 shows the parameter settings of RR and our method.

**TABLE 2:** Parameter settings.

Methods	Parameter settings
RR	The regularized parameter was searched from 0.0001 to 1
Our method	The regularized parameter λ was searched from 0.0001 to 1
MV-TSK-FS	We use the parameter settings recommended by the original references
simpleMKL	We use the parameter settings recommended by the original references
RFF-MKL	We use the parameter settings recommended by the original references
MV-L2-SVM	

### 4.2 Result analysis

The experimental results were reported from three aspects, i.e., feature selection of every single modality, comparison between single modality and multimodality in terms of AUC, and overfitting analysis in terms of the discrepancy between training and testing.

#### 4.2.1 Feature selection of every single modality

**FIGURE 4:**
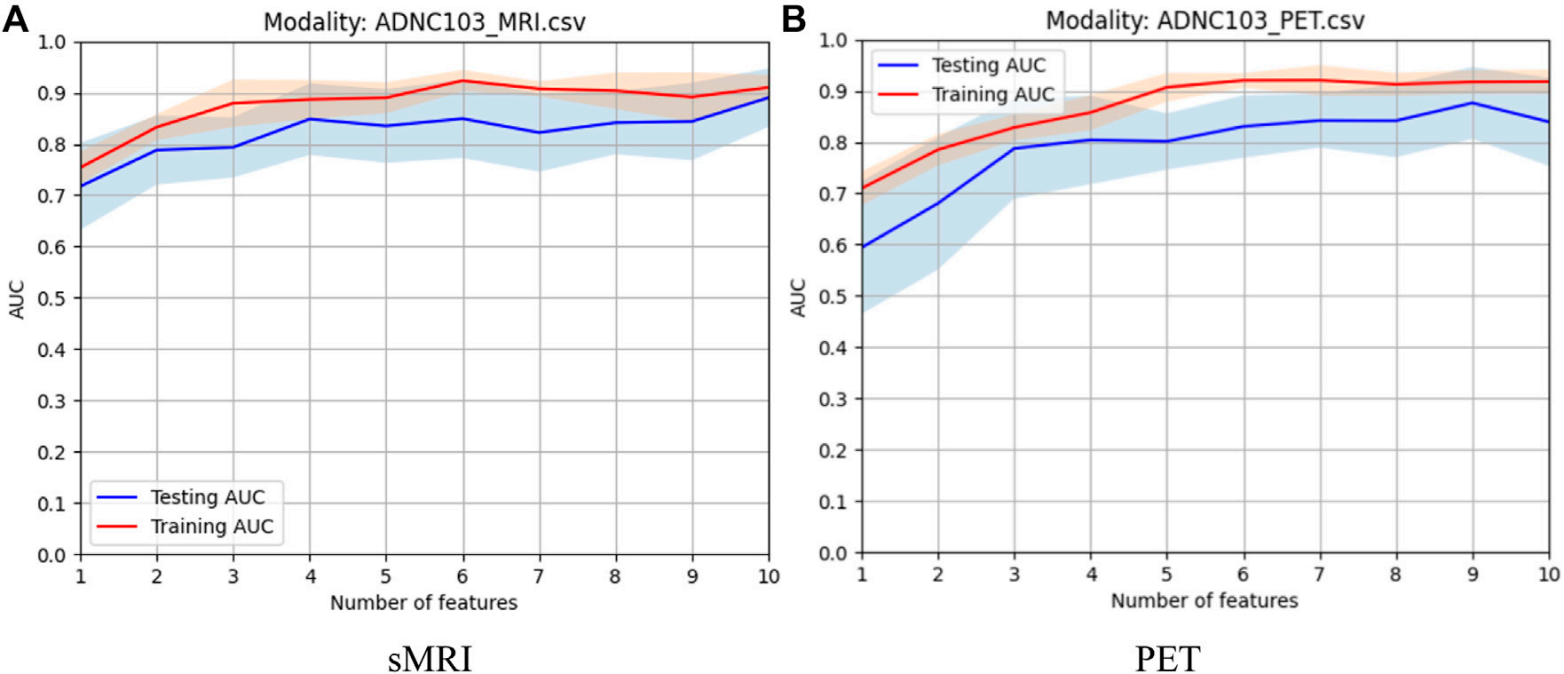
Model selection of every single modality: **(A)** sMRI and **(B)** PET.

In this study, before modality fusion, we have to select the best model for every single modality. That is to say, we should find an optimal feature subset for each modality. As we stated before, the *Fish score* was employed as the supervised method to reduce the irrelevant features to the outcome. *Person score* was employed as the unsupervised method to reduce the redundancy between features. After the two-step feature selection, we select the optimal feature set that deduces the best training AUC. As shown in Figure 4, for sMRI, it can be found that the first 6 features were selected for the following modality fusion, and for PET, the first 7 features were selected for the following modality fusion.

#### 4.2.2 Comparison between single modality and multimodality

**FIGURE 5:**
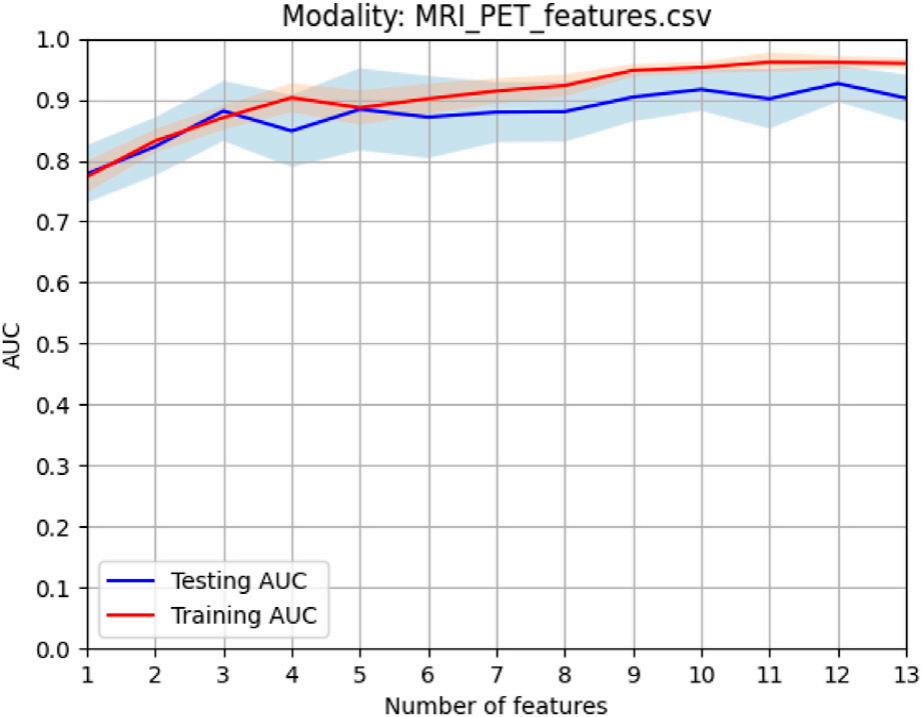
Model selection of combined features.

When the optimal feature sets of sMRI and PET were combined, feature redundancy between different modalities may also exist. Therefore, *Person score* was also employed as the unsupervised method to reduce the redundancy across different modalities. After this procedure, the best model can be obtained by finding the best training AUC. As shown in Figure 5, the first 12 features can generate the best model.

**FIGURE 6:**
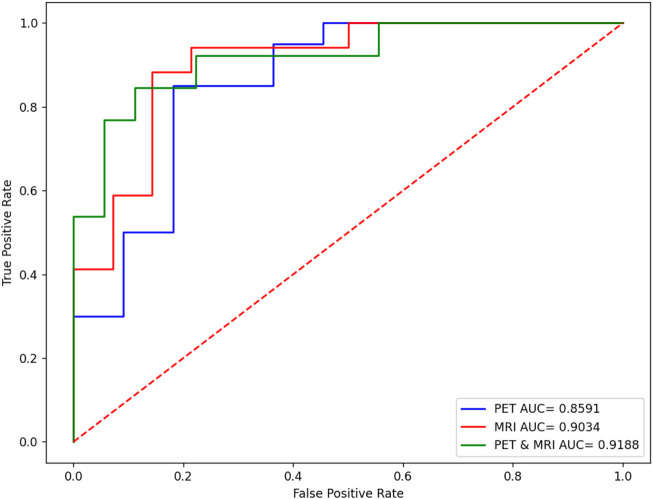
Performance comparison of sMRI, PET, and their combination.

Figure 6 shows the comparison results in terms of the ROC curve of sMRI, PET, and their combination. It can be found that the testing AUC of multimodality fusion is 0.9188, which is better than that of every single modality. This is because each modality is mapped into the kernel space and multikernel learning can explore the complementary information between the two modalities. In addition, from Eq. 10, we can see that the coefficient of the kernel matrix is sparse so that the modality which contains more patterns is endowed with more attention.

#### 4.2.3 Comparison with state-of-art multimodality methods

To highlight the promising performance of the proposed method, we introduce 4 state-of-art multimodality fusion methods for comparison studies. In addition to AUC, *accuracy* is also introduced to measure the classification performance. Table 3 shows the comparison results in terms of both accuracy and AUC, where the best results are marked in bold, and “*” means that the difference between state-of-art methods and the proposed method is significant.

**TABLE 3:** Comparison with state-of-art multimodality methods in terms of accuracy and AUC.

Methods	Accuracy	AUC
MV-TSK-FS	0.9236 ± 0.0058*	0.8897 ± 0.0032*
simpleMKL	0.9454 ± 0.0047*	0.9059 ± 0.0063*
RFF-MKL	0.9402 ± 0.0025*	0.8987 ± 0.0036*
MV-L2-SVM	0.9489 ± 0.0046*	0.9021 ± 0.0047*
Our method	**0.9586** ± **0.0032**	**0.9188** ± **0.0028**

The bold means the best performance.

From Table 3, we can find that our method achieves the best performance. In particular, simpleMKL and RFF-MKL are also multikernel-based methods, but both of them perform worse than our method. This phenomenon indicates that label relaxation and compactness graph mechanisms are useful to improve the classification performance. In addition, we see that MV-TSK-FS and MV-L2-SVM perform worse than multikernel-based methods. This is because MV-TSK-FS and MV-L2-SVM both used modality-consistent regularization to achieve multimodality fusion, which did not consider the complementary information across different modalities. With the “all-single” fusion strategy used in multikernel-based methods, every single feature and the possible combinations are all considered so that the complementary information can be fully explored.

#### 4.2.4 Overfitting analysis

**FIGURE 7:**
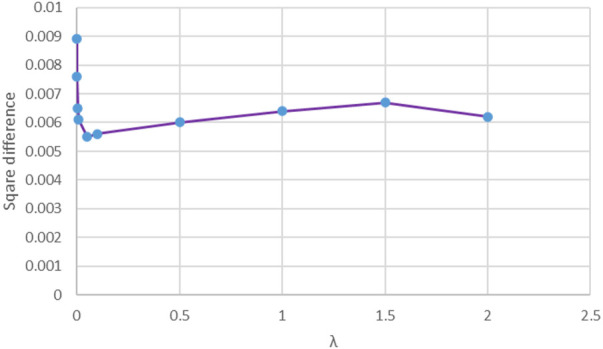
Square difference against the regularized parameter λ .

From Eq. 10, we can see that λ was used to control the contribution of the manifold regularization term. We know that the manifold regularization term can reduce overfitting; therefore, to quantificationally observe the overfitting, the square difference between training AUC and testing AUC was used. Figure 7 shows the square difference against the regularized parameter λ . From Figure 7, it can be found that from λ=0.001 to λ=0.05 , the square difference between training AUC and testing AUC decreased gradually, which means that overfitting was reduced and the generalization ability was improved. This is because the manifold regularization term in the objective function assumes that when the training samples were transformed from the feature space to the label space, if two samples are in the same manifold in the feature space, they are also in the same class the label space (Fang et al., 2017). With this assumption, sparse samples, noisy samples, or outlies will be compressed into a compact class so that the hyperplane will not excessively fit these samples.

## 5 Conclusion

In the area of personalized medicine, multimodal neuroimage data fusion plays a significant role in brain disease diagnosis. Multikernel learning actually provides a natural framework for multimodality fusion. However, when multikernel learning is applied to practice, e.g., medical data analysis, overfitting often exists. Therefore, in this study, according to RLR linear regression, we integrate label relaxation and compactness graph mechanisms into multikernel learning and propose a new multikernel learning algorithm for AD diagnosis. In the experimental study, the proposed method is evaluated in the scenario of AD diagnosis. The promising performance indicates the advantages of our method. However, from Figure 2, we can find that there are many kernel matrices generated during model training, which may consume a lot of CPU seconds and storage memory. Therefore, how to speed up the training and reduce storage memory is a hot topic in our future work.

## Data Availability

Publicly available datasets were analyzed in this study. The data are available on http://adni.loni.usc.edu/about/.
